# Whole‐exome sequencing of nevoid basal cell carcinoma syndrome families and review of Human Gene Mutation Database *PTCH1* mutation data

**DOI:** 10.1002/mgg3.498

**Published:** 2018-11-08

**Authors:** D. Matthew Gianferante, Melissa Rotunno, Michael Dean, Weiyin Zhou, Belynda D. Hicks, Kathleen Wyatt, Kristine Jones, Mingyi Wang, Bin Zhu, Alisa M. Goldstein, Lisa Mirabello

**Affiliations:** ^1^ Division of Cancer Epidemiology and Genetics National Cancer Institute, National Institutes of Health Bethesda Maryland; ^2^ Division of Cancer Control and Population Science National Cancer Institute, National Institutes of Health Bethesda Maryland; ^3^ Cancer Genomics Research Laboratory, Leidos Biomedical Research Frederick National Laboratory for Cancer Research Frederick Maryland

**Keywords:** genotype‐phenotype, nevoid basal cell carcinoma syndrome, pathogenic mutations, PTCH1, whole‐exome sequencing

## Abstract

**Background:**

Nevoid basal cell carcinoma syndrome (NBCCS) is an autosomal dominant disorder with variable expression and nearly complete penetrance. *PTCH1* is the major susceptibility locus and has no known hot spots or genotype–phenotype relationships.

**Methods:**

We evaluated 18 NBCCS National Cancer Institute (NCI) families plus *PTCH1* data on 333 NBCCS disease‐causing mutations (DM) reported in the Human Gene Mutation Database (HGMD). National Cancer Institute families underwent comprehensive genomic evaluation, and clinical data were extracted from NCI and HGMD cases. Genotype–phenotype relationships were analyzed using Fisher's exact tests focusing on mutation type and *PTCH1* domains.

**Results:**

*PTCH1* pathogenic mutations were identified in 16 of 18 NCI families, including three previously mutation‐negative families. *PTCH1* mutations were spread across the gene with no hot spot. After adjustment for multiple tests, a statistically significant genotype–phenotype association was observed for developmental delay and gross deletion–insertions (*p* = 9.0 × 10^−6^), and suggestive associations between falx cerebri calcification and all transmembrane domains (*p* = 0.002) and severe outcomes and gross deletion–insertions (*p* = 4.0 × 10^−4^).

**Conclusion:**

Overall, 89% of our NCI families had a pathogenic *PTCH1* mutation. The identification of *PTCH1* mutations in previously mutation‐negative families underscores the importance of repeated testing when new technologies become available. Additional clinical information linked to mutation databases would enhance follow‐up and future studies of genotype–phenotype relationships.

## 1 INTRODUCTION

Nevoid basal cell carcinoma, or Gorlin, syndrome (NBCCS) is an autosomal dominant multisystem disorder with nearly complete penetrance and variable expression (Hahn et al., 1996; Kimonis et al., 1997). Nevoid basal cell carcinoma syndrome has an estimated prevalence of o in 56,000 to 164,000 (Evans, Farndon, Burnell, Gattamaneni, & Birch, 1991; Kimonis et al., 1997). Patients with NBCCS have a variety of different features including multiple basal cell carcinomas (BCCs) at a young age, odontogenic keratocysts of the jaw, pits of the palms and/or soles, ectopic calcifications of the falx cerebri, medulloblastoma (Bree & Shah, 2011; Kimonis et al., 1997). The syndrome variably includes other features, such as ovarian and cardiac fibromas, rib abnormalities, skeletal malformations, and developmental delays (Bree & Shah, 2011; Kimonis et al., 1997).

The Patched 1 (*PTCH1*, OMIM 601,309) gene is the major NBCCS susceptibility locus (Chenevix‐Trench et al., 1993; Compton et al., 1994; Farndon, Del Mastro, Evans, & Kilpatrick, 1992; Gailani et al., 1992; Goldstein, Stewart, Bale, Bale, & Dean, 1994; Hahn et al., 1996; Johnson et al., 1996; Reis et al., 1992) and has been reported in 40%–88% of NBCCS cases with higher estimates closer to 90% in more recent studies (Aszterbaum et al., 1998; Boutet et al., 2003; Kato et al., 2017; Marsh, Wicking, Wainwright, & Chenevix‐Trench, 2005; Matsudate et al., 2017; Soufir et al., 2006). Familial and sporadic BCCs display loss of heterozygosity in this region, consistent with the gene being a tumor suppressor (Chenevix‐Trench et al., 1993; Gailani et al., 1992; Hahn et al., 1996). The *PTCH1* is located on chromosome 9q22.3 and codes for a large transmembrane protein that regulates the complex sonic hedgehog (SHH) signaling pathway (Bresler, Padwa, & Granter, 2016). This pathway has many important functions including a key role in development and growth (Choudhry et al., 2014). *PTCH1* acts as a negative regulator of the SHH pathway by directly inhibiting the protein smoothened (SMO). When left unopposed*,* SMO releases glioma‐associated oncogene homolog (GLI1) from Suppressor of Fused (SUFU) inhibition. GLI1 then enters the nucleus and activates oncogenic target genes (Bresler et al., 2016; Fan et al., 2008; Rimkus, Carpenter, Qasem, Chan, & Lo, 2016; Rohatgi & Scott, 2007). Germline mutations in two other genes, *SUFU* (OMIM 607035) and *PTCH2* (OMIM 603673), have also been reported to cause rare cases of NBCCS. Both genes are negative regulators of the SHH pathway (Bresler et al., 2016; Evans et al., 2017; Fan et al., 2008; Fujii et al., 2013; Pastorino et al., 2009; Rimkus et al., 2016).

The *PTCH1* mutations reported in NBCCS patients are predominantly nonsense or frameshift mutations (64%), followed by splice site mutations (13%), large insertions or deletions (12%), and missense mutations (8%; Kato et al., 2017). Copy number alterations have only rarely been reported (Kosaki et al., 2012; Matsudate et al., 2017; Morita et al., 2015). *PTCH1* is a large gene with multiple domains, including 12 transmembrane, six extracellular, five intracellular, and an N‐terminal and C‐terminal domain (Johnson et al., 1996; Lindstrom, Shimokawa, Toftgard, & Zaphiropoulos, 2006). Pathogenic mutations have been found in all domains, but most frequently in the extracellular 1, extracellular 4, and intracellular 3 domains (Lindstrom et al., 2006). Extracellular 1 and extracellular 4 domains have been suggested to be important for SHH binding (Marigo, Davey, Zuo, Cunningham, & Tabin, 1996). However, pathogenic mutations are rarely shared among unrelated families, and no specific hot spots or genotype–phenotype correlations have been identified (Evans et al., 2017; Ikemoto et al., 2017; Kato et al., 2017; Okamoto, Naruto, Kohmoto, Komori, & Imoto, 2014).

Here, we used whole‐exome sequencing to evaluate seven NBCCS families with no known disease‐related mutation to identify potential disease‐causing mutations. We assembled *PTCH1* mutation data from the above seven and 11 other NBCCS families enrolled in our NCI study plus publicly available *PTCH1* data on 333 NBCCS disease‐causing mutations (DM) reported in the Human Gene Mutation Database (HGMD; Stenson et al., 2014) to better characterize *PTCH1* NBCCS mutations. Clinical data were extracted from our NBCCS NCI families and from the published HGMD (Stenson et al., 2014) reports to evaluate genotype–phenotype correlations.

## 2 METHODS

### 2.1 Ethical compliance

This study was approved by an ethics committee, and informed consent obtained. All participants provided written consent and were recruited through an IRB‐approved protocol.

### 2.2 Study population

**Figure 1:**
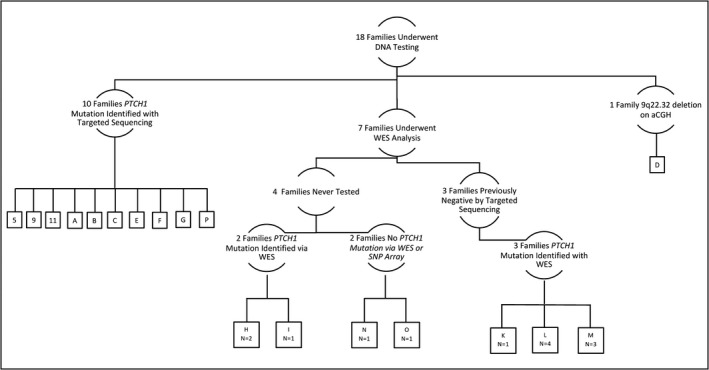
National Cancer Institute study population by *PTCH1* mutation testing type and results. Schematic of the 18 families in the NCI cohort and *PTCH1* mutation testing by DNA test type. Letters and numbers within the boxes identify different families. All families without a pathogenic *PTCH1* mutation on targeted sequencing or WES were further screened by either aCGH or SNP array to evaluate for large deletions. aCGH: array comparative genomic hybridization; SNP: single nucleotide polymorphism; WES: whole‐exome sequencing

From 1985 through the late 1990s, patients were recruited to the NCI for participation in an NBCCS clinical and gene linkage study through an NCI IRB‐approved study (Kimonis et al., 1997). Evaluation at the NCI consisted of a medical history and clinical examination, blood sample, and radiographic studies of the jaws and skull. All participants completed a detailed family and medical history questionnaire, medical records were reviewed, and a subset of eligible families underwent clinical evaluation at the NCI Clinical Center. For the current study, there were 18 families with DNA available for analysis, with a total of 62 clinical records for data extraction (Figure 1).

### 2.3 Targeted PTCH1 testing

Prior to identification of the *PTCH1* locus, families were evaluated with linkage analysis to identify carrier status of unknown NBCCS mutations. Once the *PTCH1* was identified, all family members with an NBCCS phenotype and available DNA were tested for a *PTCH1* mutation using DNA‐based sequencing. Fourteen of the families had genetic testing with targeted *PTCH1* sequencing in the 1990s, and the remaining four families in the study were never tested (Figure 1).

### 2.4 Whole‐exome sequencing and analysis

The seven families (total patients = 13) with no known mutation were included for whole‐exome sequencing (WES). This includes the three families that had no mutation identified on targeted *PTCH1* testing as well as the four families that were never tested (Figure 1). DNA was derived from previously collected blood samples. DNA samples were staged and underwent quality control analyses by the NCI Division of Epidemiology and Genetics (DCEG) DNA Extraction and Staging Laboratory (DESL).

Exome sequencing was performed at the Cancer Genomics Research (CGR) laboratory. Exome enrichment was performed with NimbleGen's SeqCap EZ Human Exome Library v3.0, targeting 64 Mb of exonic sequence on an Illumina HiSeq, as previously described (Mirabello et al., 2017, 2014 ). Briefly, “known gene” transcript annotations were downloaded from UCSC database version hg19 (GRCh37). Sequencing reads were trimmed using the Trimmomatic program (v0.32) and then aligned to the hg19 reference genome using the Novoalign software (v3.00.05). Local realignment was refined around known and novel sites of insertion and deletion polymorphisms using the RealignerTargetCreator and IndelRealigner modules from the Genome Analysis Toolkit (GATK v3.1; DePristo et al., 2011). Variant calling performed on all individuals globally using the UnifiedGenotyper and HaplotypeCaller modules from GATK as well as the FreeBayes variant caller (v9.9.2), and all three calls were then integrated using an Ensemble variant calling pipeline (v0.2.2). Exome sequencing was performed to a sufficient depth to achieve a minimum coverage of 15 reads in at least 80% of the coding sequence from the UCSC hg19 transcripts database. For the NBCCS families, 92% of coding sequence had >15 reads and the average coverage across the genome was 49 reads; for *PTCH1,* 94% of the coding sequence had >15 reads with an average of 50 reads in these families (Supporting Information Table S1). For PTCH1, we used reference sequence version NM000264.3.

Annotation of each exome variant locus was performed using a custom software pipeline developed by CGR. This pipeline adds functional annotations at the DNA level, RNA level, and protein/histone level and integrates multiple public‐domain applications including SnpEff/SnpSift ( https://snpeff.sourceforge.net/), ANNOVAR (Wang, Li, & Hakonarson, 2010; https://www.openbioinformatics.org/annovar/), and public databases such as UCSC GoldenPath database ( https://hgdownload.cse.ucsc.edu/goldenPath/hg19/database/), ESP6500 dataset from University of Washington's Exome Sequencing Project ( https://evs.gs.washington.edu/EVS/), dbNSFP—database of human nonsynonymous SNPs and function predictions (Liu, Jian, & Boerwinkle, 2011; https://sites.google.com/site/jpopgen/dbNSFP), the Molecular Signatures Database—MSigDB ( https://www.broadinstitute.org/gsea/msigdb/index.jsp), National Center for Biotechnology Information dbSNP database (Sherry et al., 2001) build 137, and 1,000 Genomes Project (Genomes Project, et al., 2010). Identified mutations of interest were validated on a different platform, using Ion Torrent, to rule out technical errors. All WES data were submitted to the publicly available database of Genotypes and Phenotypes (dbGaP; https://www.ncbi.nlm.nih.gov/gap).

### 2.5 WES bioinformatics analysis

We focused on rare mutations occurring in genes of interest. The candidate gene list was made up of all genes known to cause NBCCS (*PTCH1, PTCH2*, and *SUFU*; Bresler et al., 2016; Fan et al., 2008 *)* as well as 155 genes that directly interact with these genes in the sonic hedgehog pathway (Supporting Information Table S2). Additionally, we evaluated mutations in 114 established cancer predisposing genes (CPG) as published by Rahman (2014; Supporting Information Table S3). To aid in filtering out common/benign mutations, data from the UCSC GoldenPath database, the ESP6500 dataset from the Exome Variant Server, NHLBI Exome Sequencing Project (ESP), the Exome Aggregation Consortium (ExAC), and the 1,000 Genomes Project were used. Mutations were considered rare if the minor allele frequency (MAF) was less than 1% in these publicly available databases (ESP, ExAC, and 1,000 Genomes Project), 1,000 DCEG cancer‐free controls and an in‐house database of approximately 2,000 familial samples that underwent WES in parallel with the NBCCS families. Mutations were considered pathogenic if the mutation was categorized as high impact on the protein (i.e., nonsense, deletion, insertion, splice site) or predicted damaging in two of three in silico computer prediction models. In silico models and the damaging definition used were as follows: Combined Annotation Dependent Depletion (CADD) score >25, Meta Support Vector Machine (SVM) output of damaging, and Rare Exome Variant Ensemble Learner (REVEL) score >0.5. HGMD and ClinVar databases were used to identify if pathogenic mutations were previously reported in the literature, and data were extracted on 10 January 2017. The more recently updated *PTCH1* Leiden Open Variation Database (LOVD) in 2018 was not available for inclusion in our primary analysis (Reinders et al., 2018), although novel variants were checked for presence in this database and none were present. ClinVar was restricted to the 17 laboratories that met the minimum requirements for data sharing and quality assurance (ClinGen, 2018), and HGMD was restricted to DM.

### 2.6 Deletion analyses

All families without a pathogenic *PTCH1* mutation identified on targeted testing (*N* = 4) and underwent array comparative genomic hybridization (aCGH) for analysis of large deletions in the 1990 s using © Oxford Gene Technology. We measured genomewide germline CNVs in the four NBCCS familial cases. The CytoSure Interpret software was used to analyze the CNV data. If the identified alterations were adjacent to each other, they were further collapsed in a single CNV. Since high‐penetrance variants are likely uncommon in the general population, we filtered out CNVs with frequency larger than 5% based on information from the Toronto Database of Genomic Variants (DGV) limited to Caucasian populations only.

Genomewide single nucleotide polymorphism (SNP) microarray data were later used to evaluate large deletions in two families in which no pathogenic mutations were identified with WES and in whom aCGH testing was never done (NBCCS_N and NBCCS_O). Infinium HumanOmniExpress BeadChip technology (Illumina Inc. San Diego, CA) was performed at the NCI's CGR laboratory. Genotyping was performed according to manufacturer's guidelines using the Infinium HD Assay automated protocol. Samples were denatured and neutralized and then isothermally amplified by whole‐genome amplification. The amplified product was enzymatically fragmented and then precipitated and resuspended before hybridization to the BeadChip. Single‐base extension of the oligos on the BeadChip, using the captured DNA as a template, incorporates tagged nucleotides on the BeadChip, which were subsequently fluorophore labeled during staining. The fluorescent label determines the genotype call for the sample. The Illumina iScan scanned the BeadChips at two wavelengths to create image files.

### 2.7 NCI Family clinical data extraction

One clinician (M.G.) extracted all the clinical data from the NCI patient's charts. Data extracted included gender, basal cell carcinoma number, presence of palmoplantar pits, odontogenic keratocysts, falx cerebri calcifications, medulloblastoma, skeletal anomalies, ovarian fibromas, and developmental delay. BCC was further categorized based upon number: <50 BCC, >50 BCC, BCC present but no quantified number available, and <50 BCC but patient <40 years of age. Patients were classified according to features observed in each family and on an individual level. A family was classified as phenotypic feature present if any member had the feature. For example, if one woman in a family of two women had an ovarian fibroma, the family (and the woman) would be coded as ovarian fibroma‐positive.

### 2.8 HGMD data extraction

All *PTCH1* DM for NBCCS patients reported in HGMD (Stenson et al., 2014) were extracted for review. Data were extracted on 10 January 2017 using HGMD Professional version 2017.4. Included mutations were restricted to *PTCH1* DM reported in patients with NBCCS or Gorlin Syndrome. We excluded one family that was reported to have three different unique *PTCH1* DM. The mutation type and mutation location were recorded directly from HGMD, and all associated article(s) were downloaded from PubMed. For English language journals, all available clinical data were extracted. The same clinical features mentioned above for NCI families were classified here according to features observed in each family but not on an individual level because many publications did not provide individual‐level data.

### 2.9 Genotype–phenotype statistical analyses

All statistical analyses used family as the analysis unit. For both HGMD and NCI data, all *PTCH1* mutations were subdivided based upon *PTCH1* domain and mutation type and then analyzed for associations with patient clinical data. Specifically, each *PTCH1* domain or mutation type (missense, nonsense, small indel, gross insertion or deletion, splice) was evaluated by clinical phenotype categorized as yes/no for BCC category (present, >50, <50), falx cerebri calcification, odontogenic keratocysts, ovarian fibroma, medulloblastoma, and developmental delay. A “severe mutation” category was created as a combination of gross insertion or deletion, small deletion, small insertion, small indel, splice, and nonsense mutations. A “severe outcome” category was created and included all families who had at least one of the following phenotypes: BCC > 50, ovarian fibroma, medulloblastoma, developmental delay, and meningioma. Fisher's exact tests were used to test for significant differences by phenotype using Stata SE version 14 (StataCorp, 2015). Due to the large number of genotype–phenotype comparisons (*N* tests = 380), a Bonferroni correction for multiple tests was used and only *p*‐values <1.3 × 10^−4^ were considered statistically significant. St. Jude Cloud PeCan tool was used to create lollipop plots of *PTCH1* mutations (Zhou et al., 2016).

## 3 RESULTS

### 3.1 Characteristics of the PTCH1 mutations

*PTCH1* pathogenic mutations were identified in 89% of the NCI families, 16 of the 18 families, by either direct *PTCH1* sequencing, aCGH, or WES (Table 1). This includes three families identified with WES that were previously reported negative for *PTCH1* mutations after direct sequencing (Figure 1). No pathogenic mutations were identified in *PTCH2, SUFU*, the SHH pathway genes, or the CPG candidate gene list (Table 1; Supporting Information Table S2 and Table S3 list the SHH and CPG genes). Of the *PTCH1* mutations, 31% were frameshifts, 25% missense, 19% nonsense, 13% were inframe deletions, 6% splice sites, and 6% gross deletions. Overall, 13 of these *PTCH1* mutations have only been reported by our group, four mutations in 1996, and an additional nine mutations in the current study (Chidambaram et al., 1996; Hahn et al., 1996; Table 2). Of the three remaining *PTCH1* mutations, two were previously reported by one additional group and one was reported by two additional groups in HGMD (Fujii et al., 2003; Guo et al., 2013; Kato et al., 2017; Wicking et al., 1997; Table 2). None of these mutations were reported in ClinVar. Three of the five mutations identified with WES have not been previously reported and are considered novel. They include one frameshift, one nonsense, and one splicing mutation.

**Table 1:** Pathogenic mutations identified in the NBCCS NCI families by candidate list and mutation type

Genes or candidate lists	Mutations	Number of families	Percent of families
*PTCH1*	Frameshift	5	28
	Missense	4	22
	Nonsense	3	17
	Inframe deletion	2	11
	Gross deletion	1	6
	Splicing	1	6
	Any *PTCH1*	16	89
*SUFU*	None		
*PTCH2*	None		
SHH Candidate list	None		
CPG Candidate list	None		
Not detected		2	11

Notes

Percentage based upon a total of 18 families in the study. Pathogenic *PTCH1* mutations are either high impact (deletion, insertion, splice, nonsense) or predicted damaging by ≥2 of 3 in silico programs and have minor allele frequency <0.001% based on 1,000 Genomes Project, Exome Sequencing Project (ESP), and Exome Aggregation Consortium (ExAC) databases.

CPG: cancer predisposing genes; SHH: sonic hedgehog pathway.

**Table 2:** *PTCH1* pathogenic mutations identified in the NBCCS NCI cohort

Family ID	Method mutation identified	Genomic position mutation (GRCh37/hg19)	cDNA position mutation (NM_000264.3)	Protein alteration in nonsense and missense mutations	Previously reported references
5	Targeted Seq.	g.98239118C>G	c.1525G>C	p.Gly509Arg	Chidambaram et al., (1996)
9	Targeted Seq.	g.98229508delTGC	c.2448_2450delGCA		Chidambaram et al., (1996)
11	Targeted Seq.	g.98215814G>T	c.3395C>A	p.Ser1132Tyr	Chidambaram et al. (1996)
A	Targeted Seq.	g.98212205A>C	c.3467 T>G	p. Leu1156Arg	
B	Targeted Seq.	g.98239117C>T	c.1526G>A	p.Gly509Asp	Fujii et al. (2003) and Guo et al. (2013)
C	Targeted Seq.	g.98232202delTAC	c.1738_1740delGTA		
E	Targeted Seq.	g.98248017delG	c.534delC		
F	Targeted Seq.	g.98220509G>C	c.2954C>G	p.Ser985X	
G	Targeted Seq.	g.98221957delG	c.2812delC		
P	Targeted Seq.	g.98241404G>A	c.1093C>T	p.Gln365X	Hahn et al. (1996)
D	aCGH	27 kb deletion in 9q22.32			
H	WES	g.98218697T>G	c.3169–2A>C		
I	WES	g.98268804delG	c.279delC		Kato et al. (2017)
K	WES	g.98231085delGA	c.2197_2198delTC		Wicking et al. (1997)
L	WES	g.98224253 C>T	c.2588G>A	p.Trp863X	
M	WES	g.98244431delT	c.639delA		

Notes

Mutations are organized by the method of *PTCH1* mutation identification: targeted *PTCH1* sequencing, aCGH, and WES. All mutations listed have minor allele frequency <0.001% in 1,000 Genomes Project, Exome Sequencing Project (ESP), and Exome Aggregation Consortium (ExAC) databases and are either high impact (deletion, insertion, splice, nonsense) or predicted damaging by ≥2 of 3 in silico programs. Previously reported based on HGMD and ClinVar databases.

aCGH: array comparative genomic hybridization; Targeted Seq.: targeted sequencing; WES: whole‐exome sequencing.

**Figure 2:**
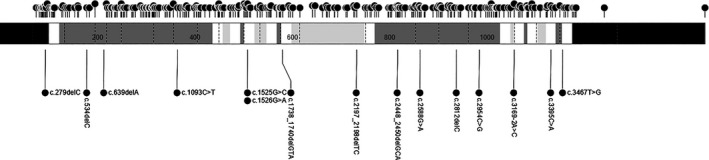
Distribution of *PTCH1* mutations by protein domains in NCI and HGMD patients. Mutations above the gene are from HGMD samples; below are mutations from NCI patients. cDNA position based on transcript NM_000264.3. Dotted lines indicate exon boundaries. Different shades of gray, white, and black represent different *PTCH1* protein domains: C/N‐terminal = black, transmembrane domains = white, extracellular = dark gray, and intracellular domains = light gray

The location of the *PTCH1* mutations, both HGMD and NCI, was spread across all domains with no apparent hotspots (Figure 2; Table 3). Most mutations were located in extracellular 1, extracellular 4, or intracellular three domains (Table 3).

**Table 3:** Distribution of *PTCH1* pathogenic mutations identified in HGMD and NCI cohort

HGMD			NCI		
Mutation/Domain	Freq.	Percent	Mutation/Domain	Freq.	Percent
Gross deletion or insertion	26	8	Gross deletion or insertion	1	6
C‐Terminal	5	2	C‐Terminal	0	0
E1	88	26	E1	3	19
E2	3	1	E2	0	0
E3	3	1	E3	0	0
E4	70	21	E4	5	31
E5	4	1	E5	0	0
E6	2	1	E6	0	0
I1	4	1	I1	0	0
I2	6	2	I2	0	0
I3	22	7	I3	1	6
I4	1	0	I4	0	0
I5	4	1	I5	0	0
T1	6	2	T1	0	0
T2	12	4	T2	0	0
T3	6	2	T3	0	0
T4	10	3	T4	2	13
T5	7	2	T5	0	0
T6	5	2	T6	1	6
T7	5	2	T7	0	0
T8	2	1	T8	0	0
T9	8	2	T9	1	6
T10	6	2	T10	0	0
T11	6	2	T11	1	6
T12	4	1	T12	1	6
N‐Terminal	15	5	N‐Terminal	0	0

Notes

Included *PTCH1* mutations are either labeled as a disease‐causing mutation (DM) by HGMD or defined as pathogenic in the NCI cohort: high impact (deletion, insertion, splice, nonsense) or predicted damaging by ≥2 of 3 in silico programs and have a minor allele frequency <0.001% based on 1,000 Genomes Project, Exome Sequencing Project (ESP), and Exome Aggregation Consortium (ExAC) databases. Three HGMD DM are not included in the above regions.

E: extracellular; I: intracellular; T: transmembrane.

### 3.2 Clinical characteristics of the NCI patients

Table 4 shows the NBCCS clinical features of the 62 NBCCS NCI patients (from 18 families) at both the family and individual levels. Family sizes ranged from 1 to 16 with a mean of 3.4 and median of 2.5. Patient gender was equal, 50% male and 50% female (Table 4). Nearly all families (*N* = 17) had at least one member with a BCC. Only family NBCCS_L had no BCCs in all four evaluated family members. All members of family NBCCS_L were >40 years of age at the time of last clinical evaluation. Across all ages, 64% of patients had <50 BCC, 23% had >50 BCC, and 13% had presence of BCC but unclear number recorded in patient medical chart. Palmoplantar pits and odontogenic keratocysts were seen in the vast majority of subjects, 89% and 92% of the families, respectively (Table 4). Falx cerebri calcification was present in 60% of the patients, but unknown in 23% due to unavailable radiologic studies. Medulloblastoma was found in 5% (*N* = 3) of patients. Among women, 19% of patients (*N* = 6) had a history of ovarian fibroma and an additional 10% (*N* = 3) had an ovarian mass on screening ultrasound at the NCI.

**Table 4:** Clinical phenotype by family and individual in the NBCCS NCI cohort

Family ID	Charts	Gender		Basal cell carcinoma				Palmoplantar pits			Odontogenic Keratocysts			Falx cerebri calcifications			Mbl.		Ovarian fibroma		
		M	F	<50	>50	<50 & age <40	Not Clear	Yes	No	Unk.	Yes	No	Unk.	Yes	No	Unk.	Yes	No	Yes	No	U/S Mass
5	2	1	1	1		1		1	1		2			2			1	1		1	
9	8	3	5	5	2		1	7		1	7		1	3		5		8	1	4	
11	1		1		1			1			1				1			1	1		
A	16	7	9	9	4	2	1	16			16			9	4	3		16	1	7	1
B	3	1	2	1	2			3			3				1	2		3		1	1
C	2	2			1	1		2			2			1	1			2			
D	2	1	1	2				2			2			2				2	1		
E	3	1	2		1	1	1	3			3			2	1			3		2	
F	3	1	2	3				3			3			3				3	1	1	
G	3	2	1			2	1	2		1	1	2		1	1	1		3		1	
H	1		1				1		1		1				1		1		1		
I	1	1			1				1		1			1				1			
K	1	1			1				1			1		1				1			
L	4	1	3	4				4			4			3	1			4		3	
M	3	2	1			2	1	3			2	1		3			1	2		1	
N	1	1					1	1			1			1				1			
O	1	1			1			1			1			1				1			
*p*	7	5	2	5		1	1	6		1	7			4		3		7		1	1
Total	62	31	31	30	14	10	8	55	4	3	57	4	1	37	11	14	3	59	6	22	3
**%**	100	50	50	48	23	16	13	89	6	5	92	6	2	60	18	23	5	95	19	71	10

Notes

Counts are the number of people in each family exhibiting indicated clinical feature and percent is based on 62 total people in the study, except for ovarian fibroma. For ovarian fibroma, counts are based on women in each family and percent is out of 31 total women in the study.

F: female; M = male; Mbl.: medulloblastoma; Not Clear: BCC present but unclear exact number; Unk.: unknown; U/S = ultrasound.

### 3.3 Genotype–phenotype relationship

A total of 333 HGMD DM *PTCH1* mutations met the criteria for inclusion in our study: HGMD categorized as NBCCS or Gorlin, HGMD DM, and one unique *PTCH1* mutation per family (Supporting Information Figure S1). Of the 333 DM, 48% (159 articles) had some clinical data available for extraction (Supporting Information Figure S1). Within these 159 articles, 73% reported data on BCC, 86% on odontogenic cysts, 44% on falx cerebri calcifications, and 8% on medulloblastoma. Ovarian fibroma was reported in 10 women (gender information was not available to allow estimation of the percent of total women).

For clinical features with sufficient families for evaluation (*N* ≥ 5), we evaluated each clinical feature by *PTCH1* domain, by groups of domains (i.e., all extracellular, all transmembrane, all intracellular), and mutation type using Fisher's exact test. A total of 380 tests were performed. A Bonferroni correction for multiple tests was used, and only *p*‐values <1.3 × 10^−4^ were considered statistically significant. There were no statistically significant associations for either *PTCH1* domain or mutation type for presence of BCC, odontogenic keratocysts, ovarian fibroma, medulloblastoma, falx cerebri calcifications, or meningioma (Supporting Information Table S4 and Table S5). The only statistically significant relationship was developmental delay and gross deletion–insertions (*p = *9.0 × 10^−6^; Table 5). Overall, two NCI families had any member with a developmental delay and 17 in HGMD; of those reported in HGMD, most of these patients had a gross deletion or insertion. The size of these gross deletion–insertions in megabytes (Mb) ranged from 15.6 to 0.003 with a mean of 6.1 and median of 4.9. Severe outcomes (presence of BCC > 50, ovarian fibroma, medulloblastoma, developmental delay, and/or meningioma) and gross deletion–insertions approached statistical significance (*p* = 4.0 × 10^−4^). Four additional associations with falx cerebri calcifications had *p*‐values <0.05: missense mutations (*p* = 0.004), severe mutations (*p* = 0.004), extracellular all (*p* = 0.045), and transmembrane all (*p* = 0.002).

**Table 5:** Association of phenotypic features by mutation type or *PTCH1* domain

Phenotype	Mutation type or domain	Number of families	*p*‐Value^c^
Developmental delay	Gross deletion or insertion^b^	8	**9.0 × 10** ^−^ **^6^**
Falx cerebri calcifications	Missense	7	0.004
	Severe mutation	76	0.004
Falx cerebri calcifications	Extracellular all	46	0.045
	Transmembrane all	16	0.002
Severe outcome^a^	Gross deletion or insertion^b^	11	4.0 × 10^−^^4^

Note

All phenotypes with a *p*‐value <0.05 and families ≥5 are included using a Fisher's exact test.

a Severe outcome category includes families with at least one of the following phenotypes: BCC > 50, ovarian fibroma, medulloblastoma, developmental delay, and meningioma.

b Exact genes deleted for gross deletion–insertions are unclear due to incomplete HGMD reporting.

c Statistically significant *p*‐values after Bonferroni correction are bolded.

## 4 DISCUSSION

We comprehensively evaluated NBCCS and *PTCH1* mutations in a total of 345 families by combining genomic data and well‐annotated clinical data of our NBCCS NCI families with manually extracted HGMD publicly available data to characterize the genotype and phenotype found in NBCCS. This is the largest study to date to assemble and characterize NBCCS *PTCH1*‐associated mutations and clinical data using targeted *PTCH1* sequencing, aCGH, SNP array data, WES, and HGMD data extraction.

The NBCCS NCI cohort includes 18 well‐characterized families, 14 were previously tested from the early 1990s, and a total of 16 (89%) had a *PTCH1* mutation detected. Previous rates of *PTCH1* mutation detection in NBCCS ranged from 40% to 70% in the early 2000s using predominantly direct *PTCH1* sequencing and aCGH (Aszterbaum et al., 1998; Boutet et al., 2003; Marsh et al., 2005; Soufir et al., 2006). Our results are more consistent with the 86%–88% *PTCH1* NBCCS mutation rates identified more recently by groups using a similar multimodality approach (Kato et al., 2017; Matsudate et al., 2017). The higher rates of *PTCH1* mutation detection are likely due to the increased sensitivity of using multiple methods that include WES. We did not identify any *SUFU* or *PTCH2* mutations in our cohort, likely due to the high rate of *PTCH1* mutations identified in these families; however, these mutations tend to make up only a minority of cases in most studies. In one recent study that included 85 patients, only one *SUFU* and one *PTCH2* mutation were identified, making up a total of 2.4% of NBCCS patients (2/85; Kato et al., 2017). Three previously tested families were negative for *PTCH1* mutations by targeted testing and aCGH but were found here to have a *PTCH1* mutation using WES. These three *PTCH1* mutations consisted of two small deletions and one nonsense mutation. This observation suggests that *PTCH1* mutations may be responsible for NBCCS in more patients/families than previously reported and underscores the importance of continued testing of unknown NBCCS families using more comprehensive approaches as new technologies become available. The two families (NBCCS_N and NBCCS_O) without a disease‐associated mutation, in *PTCH1* or other candidate genes, had no unusual NBCCS clinical phenotypes; both families had BCC, palmoplantar pits, odontogenic keratocysts, and falx cerebri calcifications.

Mutations were spread across the central gene region with extracellular 1, extracellular 4, and intracellular 3 domains housing the largest percentage of pathogenic mutations. This pattern is consistent with previous smaller studies that reported pathogenic *PTCH1* mutations most commonly in the two large extracellular domains (Guo et al., 2013; Lindstrom et al., 2006). We further showed that relatively few mutations were found in the C‐terminus and N‐terminus domains, which indicates the central portion of *PTCH1* is important. Most *PTCH1* mutations in our NCI cohort families (81%) and HGMD (95%) were private mutations. After excluding bioinformatic studies that used only previously reported data, only 5% (18/333) of HGMD *PTCH1* mutations were reported twice and only one mutation was reported three times (0.3%).

We investigated whether there were any genotype–phenotype relationships with *PTCH1* mutations by gene domains or mutation type. Consistent with previous reports (Evans et al., 2017; Ikemoto et al., 2017; Kato et al., 2017; Okamoto et al., 2014), we found no hot spots or strong genotype–phenotype relationships. There was a suggestive association between falx cerebri calcifications and all transmembrane domains, although not statistically significant using a more conservative Bonferroni correction for multiple comparison. The lack of a strong genotype–phenotype relationship despite most pathogenic mutations located in extracellular 1, extracellular 4, and intracellular 3 domains could be a function of domain size or heterogeneous function within the domains.

By mutation type, there was a statistically significant association between developmental delay and gross deletion–insertions. Severe outcome, which included mostly patients with developmental delay, and gross deletion–insertions approached statistical significance. A relationship between gross deletions and developmental delay has been previously reported (Muller et al., 2012; Yamamoto et al., 2009). This phenotype is rare in NBCCS, and other genes deleted in the area may play a role, although exact genes deleted in HGMD families is unclear due to incomplete genomic location reporting. The genotype–phenotype relationships identified have limited generalizability due to the small numbers in the NCI cohort that may not be representative of the underlying population, possible ascertainment bias since inclusion in the study was based largely on NBCCS clinical criteria, and the incomplete clinical phenotype reporting in HGMD. In addition, using family as the analysis unit does not account for heterogeneity within the family, sizes of different families, and other genetic or environmental modifiers that can affect penetrance; however, findings warrant follow‐up in additional studies.

Nevoid basal cell carcinoma syndrome is not the only autosomal dominant hereditable cancer predisposition syndrome linked to mutations in a gene with high rates of private mutations and lacking significant hot spots or strong genotype–phenotype correlations. Neurofibromatosis type 1 (NF1), a highly penetrant autosomal dominant syndrome caused by null mutations in a tumor suppressor gene (Barrea, Vaessen, Bulk, Harvengt, & Misson, 2018; Pizzo & Poplack, 2011), has also been reported to have over a 1,000 different disease‐associated *NF1* mutations with minimal hot spot or genotype–phenotype relationships (Barrea et al., 2018). However, in rare syndromes with mostly private mutations, it is difficult to confidently rule out genotype–phenotype relationships. To determine whether a specific pathogenic mutation is associated with a specific phenotype, rare diseases like NBCCS will need to aggregate information from multiple large databases to obtain significant power. From an individual patient perspective, this relationship is important to discuss in relation to potential risks of specific associated phenotypes based upon a given mutation. This has implications in not only risk assessment but also cancer screening recommendations and family planning. Well‐curated mutation databases (i.e., HGMD and ClinVar) only have access to clinical phenotype information that is published in the original manuscript.

To evaluate mutation pathogenicity, most current bioinformatic pipelines consider previously reported mutation information from databases like HGMD and ClinVar that report mutation pathogenicity scores based on variable criteria and sources of information, and this is more challenging for diseases with high rates of private mutations. For these private predicted pathogenic mutations, confirming pathogenicity may require alternative strategies, including in vitro and/or in vivo functional studies. In summary, we report an 89% *PTCH1* mutation rate in our 18 NBCCS NCI families using a comprehensive genomic approach, and no strong genotype–phenotype relationships despite the additional extraction of clinical data from 159 NBCCS families with *PTCH1* mutations reported in HGMD. Encouraging authors to include additional Supporting Information with detailed clinical information (as in our Supporting Information Table S6) would be helpful for follow‐up and future studies to better evaluate genotype–phenotype relationships in NBCCS, which could lead to better patient care.

## CONFLICT OF INTEREST

None declared.
