# Supplementary material for: Whole‐exome sequencing of nevoid basal cell carcinoma syndrome families and review of Human Gene Mutation Database PTCH1 mutation data
Source: Mol Genet Genomic Med. 2018 Nov 8;6(6):1168–80. doi: 10.1002/mgg3.498 (PMC6305672; doi:10.1002/mgg3.498)
Supplement: Supplementary file 1 [file MGG3-6-1168-s001.docx]

Supplemental Figure 1: Total *PTCH1* DM mutations in HGMD with and without clinical information for extraction


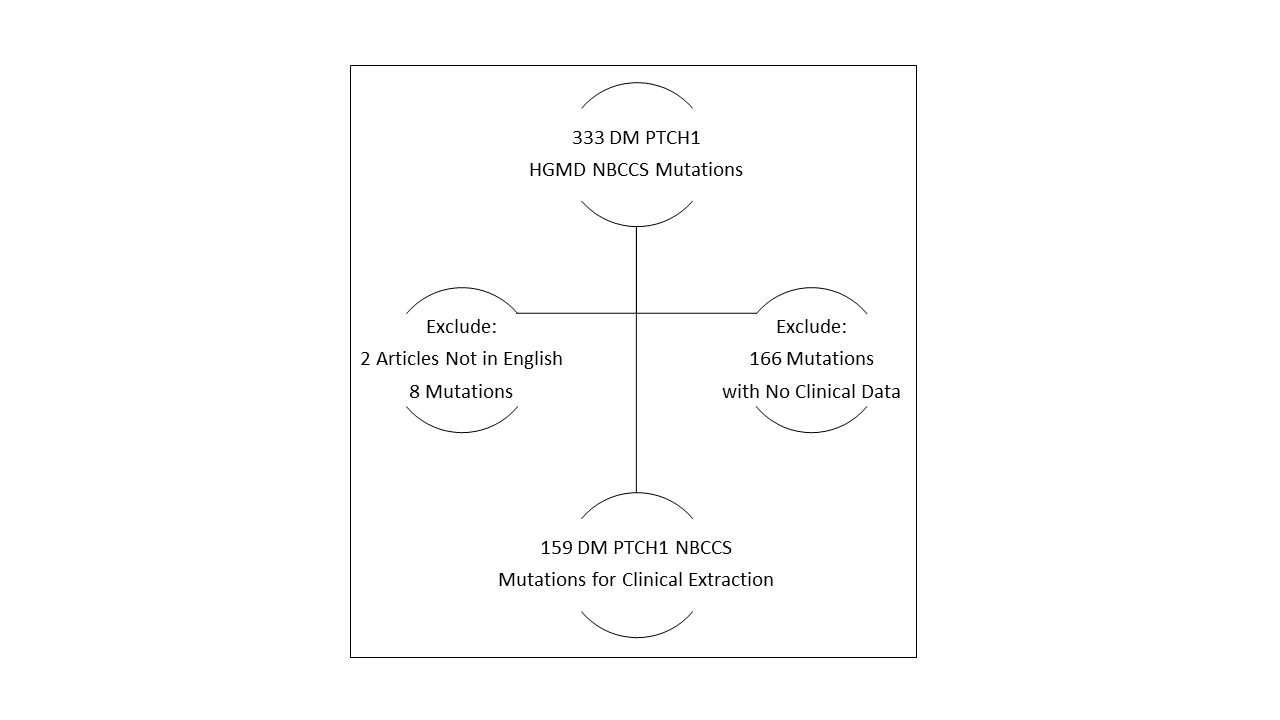


DM = disease-causing mutation.

Supplemental Table 1: Exome coverage of NBCCS patients based on UCSC transcripts

| **Analysis ID** | **% UCSC Coverage > 15X** | **UCSC Average Coverage** | **% *PTCH1* Coverage > 15X** | ***PTCH1* Average Coverage** |
| --- | --- | --- | --- | --- |
| NBCCS_H_1 | 93% | 55.48 | 97% | 55.92 |
| NBCCS_H_2 | 92% | 44.74 | 93% | 45.89 |
| NBCCS_I_1 | 93% | 51.97 | 97% | 54.96 |
| NBCCS_K_1 | 92% | 47.32 | 96% | 49.76 |
| NBCCS_L_1 | 91% | 42.18 | 95% | 50.76 |
| NBCCS_L_2 | 92% | 52.28 | 94% | 42.19 |
| NBCCS_L_3 | 93% | 53.82 | 94% | 53.5 |
| NBCCS_L_4 | 88% | 34.18 | 96% | 55.17 |
| NBCCS_M_1 | 91% | 42.71 | 94% | 34.54 |
| NBCCS_M_2 | 93% | 52.01 | 93% | 56.47 |
| NBCCS_M_3 | 92% | 48.45 | 92% | 41.97 |
| NBCCS_N_1 | 92% | 49.66 | 94% | 54.58 |
| NBCCS_O_1 | 93% | 57.32 | 94% | 48.47 |
| **Mean** | 92% | 49 | 94% | 50 |
| **Max** | 93% | 57 | 97% | 56 |
| **Min** | 88% | 34 | 92% | 35 |
| **Median** | 92% | 50 | 94% | 51 |

Analysis ID is comprised of family name and patient number separated by an underscore. Percentage is based on total University of California Santa Cruz (UCSC) coding bases.

Supplemental Table 2: Sonic hedgehog pathway candidate gene list

| SUFU | RCOR1 | GPC3 | CDKN1B | WNT4 |
| --- | --- | --- | --- | --- |
| GLIS3 | PSMD4 | SHH | CDKN1A | WNT6 |
| RNF19A | HSP90AA1 | ADRBK1 | CHEK1 | PRKACG |
| PIAS1 | RNF219 | CAV1 | PTTG1 | CSNK1A1 |
| HMGA1 | SIN3A | NEDD4 | CDC27 | WNT5B |
| ZNF219 | HDAL2 | NEDD4L | CCNA1 | WNT9A |
| ARRB2 | NOTCH1 | BMP4 | CHEK2 | PRKACB |
| UBC | AHSA1 | SMURF1 | CCNE2 | CSNK1E |
| PTCH1 | STIP1 | DHH | ANAPC4 | WNT2B |
| ULK4 | CDC37 | IHH | BUB3 | WNT16 |
| SMO | ARB2 | BMP2 | CDC25A | WNT1 |
| GLI2 | SUGT1 | PTHLH | PLK1 | PRKACA |
| HDAC2 | PSMD3 | KIF7 | HHIP | CSNK1G2 |
| RBPJ | PSMD2 | BMPR1A | DISP1 | WNT8B |
| PTCH2 | PSMD8 | CCNA2 | HHAT | WNT9B |
| STK36 | PSMC4 | APC11 | GAS1 | WNT10B |
| GLI3 | PSMD14 | FZR1 | FOXA1 | CSNK1A1L |
| HDAC1 | PSMC6 | CDC20 | CCNBB1 | WNT5A |
| GLI1 | PSMD6 | CDC25C | MAP2K1 | WNT2 |
| SAP30 | PSMC1 | LA | DERL1 | WNT7B |
| ZNF747 | RAD23A | LATS2 | DERL2 | RAB23 |
| SAP18 | PSMD12 | TEAD1 | LRP2 | CSNK1G3 |
| RBBP4 | PSMC2 | BUB1B | LRPAP1 | WNT8A |
| RBBP7 | PSMD13 | ANAPC11 | PRKCD | WNT10A |
| LAMTOR3 | PPP5C | LATS1 | BTRC | GSK3B |
| LGALS3 | PTGE53 | CDC16 | ZIC2 | CSNK1D |
| MTA2 | STIL | LENA1 | WNT3A | WNT11 |
| CHD3 | CDK1 | MAD2L1 | WNT7A | WNT3 |
| DNMT1 | CCNB1 | LLHE1 | PRKX | CCNE1 |
| KDM1A | YAP1 | RB1 | FBXW11 | PSMD7 |
| CHD4 | HIPK2 | CDK2 | CSNK1G1 | ANAPC17 |

Supplemental Table 3: List of cancer predisposing genes candidate list

| ABCB11 | FANCG | RECQL4 | CHEK2 | MUTYH | SRY |
| --- | --- | --- | --- | --- | --- |
| ALK | FH | RET | COL7A1 | NBN | STAT3 |
| APC | FLCN | RHBDF2 | CYLD | NF1 | STK11 |
| ATM | GATA2 | RMRP | DDB2 | NF2 | SUFU |
| AXIN2 | GBA | RUNX1 | DICER1 | PALB2 | TERT |
| BAP1 | GJB2 | SBDS | DIS3L2 | PDGFRA | TGFBR1 |
| BLM | GPC3 | SDHA | DKC1 | PHOX2B | TMEM127 |
| BMPR1A | HFE | SDHAF2 | DOCK8 | PMS2 | TNFRSF6 |
| BRCA1 | HMBS | SDHB | EGFR | POLD1 | TP53 |
| BRCA2 | HRAS | SDHC | ELANE | POLE | TRIM37 |
| BRIP1 | ITK | SDHD | ERCC2 | POLH | TSC1 |
| BUB1B | KIT | SERPINA1 | ERCC3 | PRKAR1A | TSC2 |
| CBL | MAX | SH2D1A | ERCC4 | PRSS1 | UROD |
| CDC73 | MEN1 | SLC25A13 | ERCC5 | PTCH1 | VHL |
| CDH1 | MET | SMAD4 | EXT1 | PTEN | WAS |
| CDK4 | MLH1 | SMARCA4 | EXT2 | PTPN11 | WRN |
| CDKN1B | MSH2 | SMARCB1 | FAH | RAD51C | WT1 |
| CDKN2A | MSH6 | SMARCE1 | FANCA | RAD51D | XPA |
| CEBPA | MTAP | SOS1 | FANCC | RB1 | XPC |

Established cancer predisposing genes (CPG) as published by Rahman et al (Rahman, 2014).

Supplemental Table 4: Association of phenotypic features by *PTCH1* domain using Fisher’s exact test

| ***PTCH1* Location** | **BCC Any** | | **BCC>50** | | **BCC<50** | | **OKC** | | | **FCC** | | **OF** | | **Mbl** | | **Dev Delay** | | **Severe** | |
| --- | --- | --- | --- | --- | --- | --- | --- | --- | --- | --- | --- | --- | --- | --- | --- | --- | --- | --- | --- |
|  | **N** | **P** | **N** | **P** | **N** | **P** | **N** | **P** | **N** | | **P** | **N** | **P** | **N** | **P** | **N** | **P** | **N** | **P** |
| **E1** | 28 | 0.295 |  |  |  |  | 36 | 0.439 | 22 | | 0.304 |  |  | 7 | 0.133 |  |  | 16 | 0.388 |
| **E4** | 26 | 0.69 |  |  |  |  | 32 | 0.525 | 21 | | 0.324 |  |  |  |  |  |  | 11 | 0.235 |
| **E1_E4** | 54 | 0.295 | 5 | 0.133 | 8 | 0.133 | 68 | 0.565 | 43 | | 0.061 | 8 | 0.62 | 9 | 0.521 | 7 | 0.201 | 27 | 0.416 |
| **E_All** | 57 | 0.255 | 5 | 0.064 | 9 | 0.064 | 73 | 0.515 | 46 | | 0.045 | 9 | 0.5 | 9 | 0.521 | 7 | 0.134 | 28 | 0.333 |
| **I3** |  |  |  |  |  |  | 10 | 0.204 | 5 | | 0.786 |  |  |  |  |  |  |  |  |
| **I_All** | 10 | 0.674 |  |  |  |  | 15 | 0.287 | 7 | | 0.711 |  |  |  |  |  |  | 5 | 0.334 |
| **T2** |  |  |  |  |  |  | 6 | 0.885 |  | |  |  |  |  |  |  |  |  |  |
| **T4** | 6 | 0.793 |  |  |  |  |  |  |  | |  |  |  |  |  |  |  |  |  |
| **T_All** | 27 | 0.292 | 6 | 0.155 |  |  | 41 | 0.384 | 16 | | 0.002 | 5 | 0.587 |  |  |  |  | 13 | 0.37 |
| **Nterm** |  |  |  |  |  |  | 6 | 0.885 | 5 | | 0.786 |  |  |  |  |  |  |  |  |

Table includes all phenotypes and all P-values with families ≥5. Gray indicates families <5 in that domain. Meningioma was excluded because families <5 in all domains. E=extracellular; I=intracellular; T=transmembrane; Nterm=n-terminal; BCC=basal cell carcinoma; OKC=odontogenic keratocysts; FCC=falx cerebri calcifications; Mbl=medulloblastoma; Dev Delay=developmental delay; Severe=severe outcome (families with at least one of the following phenotypes: BCC>50, ovarian fibroma, medulloblastoma, developmental delay, and meningioma); N=number of families; P=P-value.

Supplemental Table 5: Association of phenotypic features by *PTCH1* mutation type using Fisher’s exact test

| **Mutation Type** | **BCC Any** | | **BCC >50** | | **BCC <50** | | **OKC** | | **FCC** | | **OF** | | **Mbl** | | **Dev Delays** | | **Meng** | | **Severe** | |
| --- | --- | --- | --- | --- | --- | --- | --- | --- | --- | --- | --- | --- | --- | --- | --- | --- | --- | --- | --- | --- |
|  | N | P | N | P | N | P | N | P | N | P | N | P | N | P | N | P | N | P | N | P |
| **Gross Del or Ins** | 6 | 0.238 |  |  |  |  | 12 | 0.238 | 9 | 0.641 |  |  |  |  | **8** | **9.0x10-6** |  |  | 11 | 4.0x10^-4^ |
| **Splice** | 13 | 0.594 |  |  |  |  | 21 | 0.636 | 9 | 0.392 |  |  |  |  |  |  |  |  | 6 | 0.288 |
| **Missense** | 21 | 0.198 | 5 | 0.258 |  |  | 28 | 0.538 | 7 | 0.004 |  |  |  |  |  |  |  |  | 11 | 0.476 |
| **Nonsense** | 15 | 0.479 |  |  | 5 | 0.056 | 19 | 0.349 | 14 | 0.489 |  |  |  |  |  |  |  |  | 5 | 0.116 |
| **Small Deletion** | 34 | 0.215 | 6 | 0.155 |  |  | 43 | 0.647 | 26 | 0.234 |  |  | 6 | 0.391 |  |  |  |  | 16 | 0.419 |
| **Small Insertion** | 13 | 0.594 |  |  |  |  | 24 | 0.593 | 17 | 0.412 |  |  |  |  |  |  |  |  | 9 | 0.526 |
| **Severe Mutation** | 83 | 0.198 | 10 | 0.258 | 11 | 0.258 | 120 | 0.538 | 76 | 0.004 | 13 | 0.668 | 12 | 0.479 | 18 | 0.107 | 7 | 0.244 | 47 | 0.476 |

Table includes all phenotypes and all P-values with families ≥5. Gray indicates families <5 in that mutation type. Small Indel was excluded because all families <5. Statistically significant P-values after Bonferonni correction are bolded. BCC=basal cell carcinoma; OKC=odontogenic keratocysts; FCC=falx cerebri calcifications; Mbl=medulloblastoma; Dev Delay=Developmental Delay; Meng=meningioma; Severe=severe outcome (families with at least one of the following phenotypes: BCC>50, ovarian fibroma, medulloblastoma, developmental delay, and meningioma); N=number of families; P=P-value; Gross Del or Ins=Gross deletion or insertion; Severe Mutation=combination of gross deletion or insertion, small deletion, small insertion, small indel, splice, and nonsense mutations.

Supplemental Table 6: Clinical data for NBCCS patients from each NCI family

| **ID** | **Pathogenic *PTCH1* Genomic Location (GRCh37/hg19)** | **Gender** | **BCC** | **PPP** | **OKC** | **FCC** | **OF** | **Mbl** | **DD** | **Meng** |
| --- | --- | --- | --- | --- | --- | --- | --- | --- | --- | --- |
| NBCCS_5_1 | g.98239118C>G | 1 | 10 | 1 | 1 | 1 | 0 | 1 | 1 | 0 |
| NBCCS_5_2 | g.98239118C>G | 2 | 0 | 0 | 1 | 1 | 0 | 0 | 0 | 0 |
| NBCCS_9_1 | g.98229508delTGC | 1 | 2 | 1 | 9 | 9 | 0 | 0 | 0 | 0 |
| NBCCS_9_2 | g.98229508delTGC | 2 | 1 | 1 | 1 | 9 | 0 | 0 | 0 | 0 |
| NBCCS_9_3 | g.98229508delTGC | 2 | 3 | 9 | 1 | 1 | 1 | 0 | 0 | 0 |
| NBCCS_9_4 | g.98229508delTGC | 1 | 2 | 1 | 1 | 1 | 0 | 0 | 0 | 0 |
| NBCCS_9_5 | g.98229508delTGC | 2 | 1 | 1 | 1 | 9 | 0 | 0 | 0 | 0 |
| NBCCS_9_6 | g.98229508delTGC | 2 | 1 | 1 | 1 | 1 | 0 | 0 | 0 | 0 |
| NBCCS_9_7 | g.98229508delTGC | 1 | 1 | 1 | 1 | 9 | 0 | 0 | 0 | 0 |
| NBCCS_9_8 | g.98229508delTGC | 2 | 1 | 1 | 1 | 9 | 0 | 0 | 0 | 0 |
| NBCCS_11_1 | g.98215814G>T | 2 | 2 | 1 | 1 | 0 | 1 | 0 | 0 | 0 |
| NBCCS_A_1 | g.98212205A>C | 2 | 1 | 1 | 1 | 1 | 0 | 0 | 0 | 0 |
| NBCCS_A_2 | g.98212205A>C | 2 | 1 | 1 | 1 | 1 | 2 | 0 | 0 | 0 |
| NBCCS_A_3 | g.98212205A>C | 1 | 1 | 1 | 1 | 1 | 0 | 0 | 0 | 0 |
| NBCCS_A_4 | g.98212205A>C | 1 | 2 | 1 | 1 | 9 | 0 | 0 | 0 | 0 |
| NBCCS_A_5 | g.98212205A>C | 2 | 1 | 1 | 1 | 1 | 0 | 0 | 0 | 0 |
| NBCCS_A_6 | g.98212205A>C | 2 | 1 | 1 | 1 | 0 | 0 | 0 | 0 | 0 |
| NBCCS_A_7 | g.98212205A>C | 1 | 2 | 1 | 1 | 1 | 0 | 0 | 0 | 0 |
| NBCCS_A_8 | g.98212205A>C | 2 | 1 | 1 | 1 | 1 | 0 | 0 | 0 | 0 |
| NBCCS_A_9 | g.98212205A>C | 2 | 2 | 1 | 1 | 1 | 0 | 0 | 0 | 0 |
| NBCCS_A_10 | g.98212205A>C | 2 | 2 | 1 | 1 | 0 | 1 | 0 | 0 | 0 |
| NBCCS_A_11 | g.98212205A>C | 1 | 10 | 1 | 1 | 0 | 0 | 0 | 0 | 0 |
| NBCCS_A_12 | g.98212205A>C | 1 | 10 | 1 | 1 | 0 | 0 | 0 | 0 | 0 |
| NBCCS_A_13 | g.98212205A>C | 1 | 3 | 1 | 1 | 1 | 0 | 0 | 0 | 0 |
| NBCCS_A_14 | g.98212205A>C | 2 | 1 | 1 | 1 | 1 | 0 | 0 | 0 | 0 |
| NBCCS_A_15 | g.98212205A>C | 2 | 1 | 1 | 1 | 9 | 0 | 0 | 0 | 0 |
| NBCCS_A_16 | g.98212205A>C | 1 | 1 | 1 | 1 | 9 | 0 | 0 | 0 | 0 |
| NBCCS_B_1 | g.98239117 C>T | 2 | 1 | 1 | 1 | 9 | 0 | 0 | 0 | 0 |
| NBCCS_B_2 | g.98239117 C>T | 2 | 2 | 1 | 1 | 0 | 2 | 0 | 0 | 0 |
| NBCCS_B_3 | g.98239117 C>T | 1 | 2 | 1 | 1 | 9 | 0 | 0 | 0 | 0 |
| NBCCS_C_1 | g.98232202delTAC | 1 | 2 | 1 | 1 | 0 | 0 | 0 | 0 | 0 |
| NBCCS_C_2 | g.98232202delTAC | 1 | 10 | 1 | 1 | 1 | 0 | 0 | 0 | 0 |
| NBCCS_D_1 | 9q22.32 deletion | 1 | 1 | 1 | 1 | 1 | 0 | 0 | 0 | 0 |
| NBCCS_D_2 | 9q22.32 deletion | 2 | 1 | 1 | 1 | 1 | 1 | 0 | 0 | 0 |
| NBCCS_E_1 | g.98248017delG | 1 | 2 | 1 | 1 | 1 | 0 | 0 | 0 | 0 |
| NBCCS_E_2 | g.98248017delG | 2 | 3 | 1 | 1 | 0 | 0 | 0 | 0 | 0 |
| NBCCS_E_3 | g.98248017delG | 2 | 10 | 1 | 1 | 1 | 0 | 0 | 0 | 0 |
| NBCCS_F_1 | g.98220509G>C | 2 | 1 | 1 | 1 | 1 | 0 | 0 | 0 | 0 |
| NBCCS_F_2 | g.98220509G>C | 1 | 1 | 1 | 1 | 1 | 0 | 0 | 0 | 0 |
| NBCCS_F_3 | g.98220509G>C | 2 | 1 | 1 | 1 | 1 | 1 | 0 | 0 | 0 |
| NBCCS_G_1 | 98221957delG | 1 | 3 | 9 | 1 | 9 | 0 | 0 | 0 | 0 |
| NBCCS_G_2 | 98221957delG | 2 | 10 | 1 | 0 | 1 | 0 | 0 | 0 | 0 |
| NBCCS_G_3 | 98221957delG | 1 | 10 | 1 | 0 | 0 | 0 | 0 | 0 | 0 |
| NBCCS_H_1 | g.98218697 T>G | 2 | 3 | 0 | 1 | 0 | 1 | 1 | 1 | 0 |
| NBCCS_I_1 | g.98268804delG | 1 | 2 | 0 | 1 | 1 | 0 | 0 | 0 | 0 |
| NBCCS_K_1 | g.98231085delGA | 1 | 2 | 0 | 0 | 1 | 0 | 0 | 0 | 0 |
| NBCCS_L_1 | g.98224253 C>T | 2 | 0 | 1 | 1 | 1 | 0 | 0 | 0 | 0 |
| NBCCS_L_2 | g.98224253 C>T | 2 | 0 | 1 | 1 | 0 | 0 | 0 | 0 | 0 |
| NBCCS_L_3 | g.98224253 C>T | 2 | 0 | 1 | 1 | 1 | 0 | 0 | 0 | 0 |
| NBCCS_L_4 | g.98224253 C>T | 1 | 0 | 1 | 1 | 1 | 0 | 0 | 0 | 0 |
| NBCCS_M_1 | g.98244431delT | 1 | 3 | 1 | 1 | 1 | 0 | 1 | 0 | 0 |
| NBCCS_M_2 | g.98244431delT | 1 | 10 | 1 | 1 | 1 | 0 | 0 | 0 | 0 |
| NBCCS_M_3 | g.98244431delT | 2 | 10 | 1 | 0 | 1 | 0 | 0 | 0 | 0 |
| NBCCS_N_1 | none | 1 | 3 | 1 | 1 | 1 | 0 | 0 | 0 | 0 |
| NBCCS_O_1 | none | 1 | 2 | 1 | 1 | 1 | 0 | 0 | 0 | 0 |
| NBCCS_P_1 | g.98241404G>A | 2 | 3 | 1 | 1 | 1 | 2 | 0 | 0 | 0 |
| NBCCS_P_2 | g.98241404G>A | 2 | 1 | 9 | 1 | 1 | 0 | 0 | 0 | 0 |
| NBCCS_P_3 | g.98241404G>A | 1 | 1 | 1 | 1 | 9 | 0 | 0 | 0 | 0 |
| NBCCS_P_4 | g.98241404G>A | 1 | 10 | 1 | 1 | 1 | 0 | 0 | 0 | 0 |
| NBCCS_P_5 | g.98241404G>A | 1 | 1 | 1 | 1 | 1 | 0 | 0 | 0 | 0 |
| NBCCS_P_6 | g.98241404G>A | 1 | 0 | 1 | 1 | 9 | 0 | 0 | 0 | 0 |
| NBCCS_P_7 | g.98241404G>A | 1 | 0 | 1 | 1 | 9 | 0 | 0 | 0 | 0 |

ID is comprised of family name and patient number separated by an underscore. For all fields except gender and basal cell carcinoma (BCC), 0 and 1 indicates absence and presence of indicated phenotype respectively and 9 indicates unknown phenotype status. For gender: 1=male and 2=female. For BCC: 0=no BCC, 1= >1 BCC but <50 BCC, 2= >50 BCC, 3=BCC present but unclear number, 10= <50 BCC but <40 years of age at examination. BCC=basal cell carcinoma; PPP=plantar/palmar pits; OKC= odontogenic keratocysts; FCC=falx cerebri calcifications; OF=ovarian fibroma; Mbl=medulloblastoma; DD=developmental delay; Meng=meningioma.
